# Segmental isotopic labeling of a 140 kDa dimeric multi-domain protein CheA from *Escherichia coli* by expressed protein ligation and protein *trans*-splicing

**DOI:** 10.1007/s10858-012-9628-3

**Published:** 2012-06-28

**Authors:** Yuichi Minato, Takumi Ueda, Asako Machiyama, Ichio Shimada, Hideo Iwaï

**Affiliations:** 1 Graduate School of Pharmaceutical Sciences, University of Tokyo, Hongo, Bunkyo-ku, Tokyo, 113-0033 Japan; 2 Biomedicinal Information Research Center, National Institute of Advanced Industrial Science and Technology, Aomi, Koto-ku, Tokyo, 135-0064 Japan; 3 Research Program in Structural Biology and Biophysics, Institute of Biotechnology, University of Helsinki, P.O. Box 65, 00014 Helsinki, Finland

**Keywords:** Segmental isotopic labeling, Intein, Protein splicing, Expressed protein ligation, CheA, Protein *trans*-splicing

## Abstract

Segmental isotopic labeling is a powerful labeling tool to facilitate NMR studies of larger proteins by not only alleviating the signal overlap problem but also retaining features of uniform isotopic labeling. Although two approaches, expressed protein ligation (EPL) and protein *trans*-splicing (PTS), have been mainly used for segmental isotopic labeling, there has been no single example in which both approaches have been directly used with an identical protein. Here we applied both EPL and PTS methods to a 140 kDa dimeric multi-domain protein *E. coli* CheA, and successfully produced the ligated CheA dimer by both approaches. In EPL approach, extensive optimization of the ligation sites and the conditions were required to obtain sufficient amount for an NMR sample of CheA, because CheA contains a dimer forming domain and it was not possible to achieve high reactant concentrations (1–5 mM) of CheA fragments for the ideal EPL condition, thereby resulting in the low yield of segmentally labelled CheA dimer. PTS approach sufficiently produced segmentally labeled ligated CheA in vivo as well as in vitro without extensive optimizations. This is presumably because CheA has self-contained domains connected with long linkers, accommodating a seven-residue mutation without loss of the function, which was introduced by PTS to achieve the high yield. PTS approach was less laborious than EPL approach for the routine preparation of segmentally-isotope labeled CheA dimer. Both approaches remain to be further developed for facilitating preparations of segmental isotope-labelled samples without extensive optimizations for ligation.

**Electronic supplementary material:**

The online version of this article (doi:10.1007/s10858-012-9628-3) contains supplementary material, which is available to authorized users.

## Introduction

**Fig. 1:**
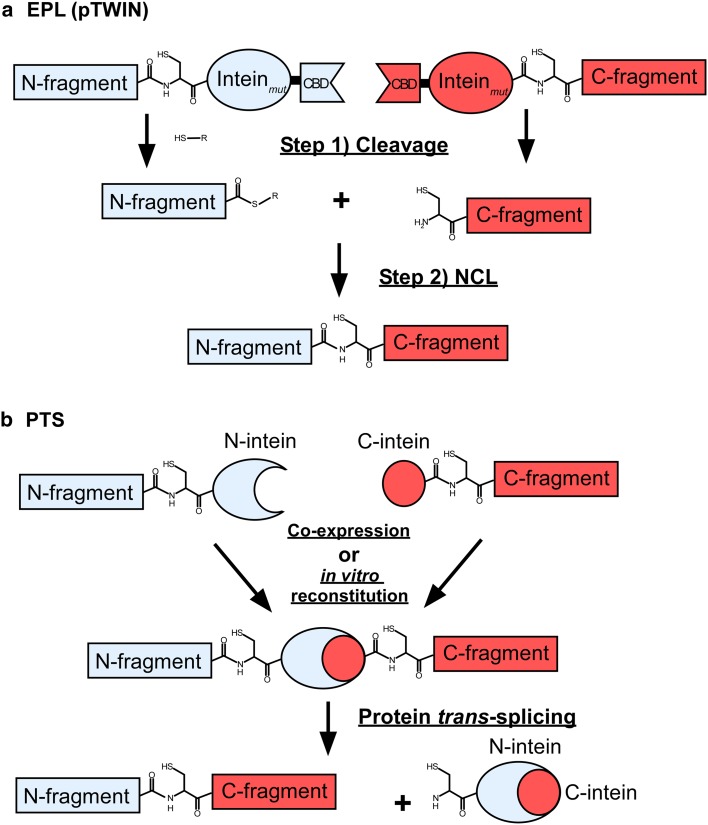
Schematic drawings for segmental isotopic labeling steps by EPL with pTWIN system (**a**) and PTS (**b**)

Stable isotopic labeling is almost prerequisite for any NMR analysis of proteins. Uniformly labeled proteins have been routinely prepared from bacterial expression systems, thereby significantly advancing NMR analysis of biomolecules by expanding dimensions of NMR spectra with triple-resonance experiments. The number of correlation peaks with larger proteins increases exponentially, enhancing the chance of signal overlaps. This signal overlap problem is an inherent problem associated with larger molecules, which is still difficult to be resolved even at the highest magnetic field currently available. There has been much effort towards reducing signal overlaps since the beginning of biomolecular NMR (Ohki and Kainosho 2008). In addition to uniform isotopic labeling, selective amino acid labeling has been used to reduce the number of NMR signals. Currently, it is feasible to introduce very sophisticated labeling schemes such as stereo-array isotope labeling (SAIL) and site-specific labeling although these techniques may not be readily accessible to non-experts (Kainosho et al. 2006; Ohki and Kainosho 2008; Jones et al. 2010). Simple and inexpensive labeling techniques without special expertise such as cell-free protein synthesis or special reagents could facilitate NMR studies of larger proteins. Selective amino acid labeling can be easily produced by feeding labeled amino acids into growth media. However, selective amino acid labeling still leaves the problem of sequence specific resonance assignments. Additional labeling such as amino acid type-selective ^13^C- and ^15^N-double labeling is required for the assignments (Ohki and Kainosho 2008). One of the promising approaches to solve both signal overlap problem and sequence specific assignments is segmental isotopic labeling, where only a region of a protein is isotopically enriched. Segmental isotopic labeling cannot only supress unwanted signals but also retain the features of uniformly labeled samples (Yamazaki et al. 1998; Xu et al. 1999; Iwai and Züger 2007; Skrisovska et al. 2010). Hence, segmental isotopic labeling could be a powerful labeling technique to investigate larger proteins, proteins bearing recurring modular domains and/or highly disordered regions by NMR spectroscopy (Busche et al. 2009). Despite its potential, examples of segmental isotopic labeling are still scarce. This is presumably because of technical hurdles associated with segmental isotopic labeling procedures to produce a large quantity for NMR studies in various labelling formats. Two major approaches have been mainly used for segmental isotopic labeling, i.e. expressed protein ligation (EPL) and protein *trans*-splicing (PTS) (Fig. 1).

EPL is based on native chemical ligation (NCL), in which one peptide with a C-terminal thioester group and the other peptide with an N-terminal cysteine are chemo-selectively ligated in aqueous solution (Fig. 1a) (Dawson et al. 1994). In EPL, two protein fragments are expressed and purified separately from bacterial expression systems, therefore allowing one of the fragments to be isotopically enriched with NMR active nuclei such as ^13^C and ^15^N (Evans et al. 1998; Xu et al. 1999). Alternatively, one of the two fragments could be prepared by chemical synthesis for the ligation, enabling introductions of selectively modified amino acids via EPL (Frutos et al. 2010). The two fragments can be ligated chemo-selectively via NCL in vitro. The requirements for NCL are an N-terminal fragment with α-thioester group at the C-terminus and an N-terminal cysteine in the C-terminal fragment. Therefore, intein-mediated protein purification has been utilized for producing thioester-modified fragments (Chong et al. 1997; Evans et al. 1998; Evans et al. 1999). On the other hand, PTS approach directly exploits protein splicing catalysed by intein. Protein splicing is a self-catalytic process in which an intervening sequence termed intein removes itself from the host precursor and concomitantly ligates the two flanking protein sequences termed exteins into one polypeptide chain. An intein-containing precursor can be split into two fragments within the intein sequence. Reconstitution of the two split precursor fragments could induce ligation of the two flanking sequences into one polypeptide chain via protein splicing in *trans* (Southworth et al. 1998; Paulus 2000) (Fig. 1b). Segmentally isotopic labeling has been demonstrated by exploiting PTS (Yamazaki et al. 1998; Otomo et al. 1999a; Yagi et al. 2004). However, the original PTS procedure with PI-*Pfu*I intein required refolding of precursor fragments, thereby making the procedure labour-intensive and limiting its application to proteins that can be refolded (Otomo et al. 1999a). Recently, PTS without refolding has been developed by exploiting naturally occurring split inteins (Züger and Iwai 2005; Muona et al. 2008; Buchinger et al. 2010). Segmental isotopic labeling using PTS without refolding has been extended to three-fragment ligation (Busche et al. 2009). PTS approach with refolding utilizing artificially split inteins such as PI-*Pfu*I and PI-*Pfu*II inteins is probably suitable for segmental isotopic labeling of a globular protein that can be refolded in vitro, because split fragments of a globular domain is unlikely to be soluble and structured (Otomo et al. 1999a, b; Yagi et al. 2004). On the other hand, PTS without refolding can be more easily used for ligation of self-contained domains connected with flexible linkers by in vivo and in vitro protocols (Muona et al. 2010).

Both EPL and PTS approaches have been used for producing segmentally labeled proteins for NMR studies (reviewed in Iwai and Züger 2007 and Skrisovska et al. 2010). Both methods have pros and cons. Because there is no single example that has been produced by both approaches, it is unclear what are the crucial points that could constrain successful production of segmentally labeled samples, particularly for large multi-domain proteins like *E. coli* CheA.

In this article, we report the production of a segmentally labeled 140 kDa dimeric multi-domain protein, *E. coli* CheA by EPL and PTS approaches. To our knowledge, this is the first example where an identical protein was produced as segmentally labeled forms by both EPL and PTS approaches, shedding light on the limiting steps in each approach.

## Materials and methods

### Cloning of CheA P1 and P2–5 domains for the EPL approach

All genes of CheA domains were amplified from genomic DNA of *E. coli* strain BL21 by PCR reactions. The genes of CheA P1 domains (residues from M3 to K146, Q153, R155, or Q157) were ligated into the vector pTWIN1 (New England Biolabs) after digestion with *Nde*I and *Sap*I, resulting in *Mxe* GyrA intein fusions (Evans et al. 1999). It is also noteworthy that the same construct could be constructed with pTXB1 (New England Biolabs). The first two residues of CheA (M1 and S2) were inserted just before the *Nde*I digestion site using QuikChange^®^ site-directed mutagenesis kit (STRATAGENE). In the following, we refer to these constructs as P1(K146), P1(Q153), P1(R155), and P1(Q157). A glycine mutation was also introduced at the C-terminus of P1(Q153) using QuikChange^®^ kit, which is referred to as P1(Q153G) unless otherwise described. The genes of CheA P2–5 domains (residues from S147, S154, S156, or S158 to A654) with N-terminal cysteine mutations at their N-termini were ligated into pTWIN1 after digestion with *Sap*I and *Bam*HI, resulting in *Ssp* DnaB intein fusions. In the following, we refer to these cysteine mutants of P2–5 domains as (S147C)P2–5, (S154C)P2–5, (S156C)P2–5, and (S158C)P2–5.

### Cloning of CheA P12 and P3–5 domains for the EPL approach

The genes of CheA P12 domains (residues from M3 to L240, A241, Q244, G248 or R257) with glycine mutations at their C-terminal residues were ligated into pTWIN1 after digestion with *Nde*I and *Sap*I, to produce *Mxe* GyrA intein fusions (Fig. 3a) and the first two residues of CheA were inserted before the *Nde*I digestion site using QuikChange^®^ protocol. In the following, we refer to these P12 domains with glycine residues at their C-termini as P12(L240G), P12(A241G), P12(Q244G), P12(G248), and P12(R257G), respectively, unless otherwise described. The genes of CheA P3–5 domains (residues from A241, A242, A245, R249, or S258 to A654) with N-terminal cysteine mutations at their N-termini were ligated into pTWIN1 after digestion with *Sap*I and *Bam*HI, resulting in the N-terminal *Ssp* DnaB intein fusions (Fig. 3b). In the following, we refer to these cysteine mutants of P3–5 domains as (A241C)P3–5, (A242C)P3–5, (A245C)P3–5, (R249C)P3–5, and (S258C)P3–5.

### Preparation of P1 and P2–5 domains and their in vitro ligation reactions

The plasmids encoding P1 or P2–5 domains were transformed to *E. coli* strain BL21-CodonPlus (DE3)-RP (STRATAGENE). The cells were grown to mid-log phase in Luria–Bertani (LB) medium, and the protein expression was induced by the addition of isopropyl-β-D-thiogalactopyranoside (IPTG) to a final concentration of 1 mM. After shaking incubation at 25 °C for 6 h, the cells were harvested and re-suspended in buffer A (20 mM Hepes–NaOH (pH 7.4), 500 mM NaCl). Subsequently, the cells were lysed by ultrasonication, and the supernatant after centrifugation were subjected to 15% SDS-PAGE analysis. The cell lysates overexpressing P1(Q153G) or (S154C)P2–5 were loaded onto chitin beads (New England Biolabs) equilibrated with buffer A, which does not contain any reducing agents. The column was washed with buffer A, and quickly soaked with 2/3 column volume (CV) of buffer B (500 mM Hepes–NaOH (pH 7.4), 100 mM NaCl, 1 mM EDTA, 200 mM 2-mercaptoethane sulfonate (MESNA)). The column was incubated at room temperature for more than 12 h. The spliced P1(Q153G) (~40 mg/L culture) or (S154C)P2–5 (~20 mg/L culture) were eluted with Bufffer A, and the buffer was exchanged to buffer B. Excess molar amounts of P1(Q153G) (6.4 mg, a final concentration of 1.5 mM) were added to (S154C)P2–5 (4.1 mg, a final concentration of 0.3 mM), and concentrated to 250 μL using a centrifugal concentrator (Amicon Ultra-15, Millipore). We used excess of P1 over P2–5 because of higher solubility of P1 and for easier purification of ligated CheA homodimer from CheA/P2–5 heterodimer in the subsequent purification. The reaction mixture in buffer B was incubated at room temperature with gentle shaking for more than 1 day. The ligation efficiency was about 40%. The reaction mixture was diluted to a final volume of 5 mL with TEDG buffer (50 mM Tris (pH 7.5), 0.5 mM EDTA, 2 mM DTT, 10% (w/v) glycerol) containing 150 mM NaCl, and loaded onto HiLoad 16/60 Superdex 200 prep grade column (GE Healthcare) and eluted with the same buffer. The fractions containing the ligated products of P1(Q153G)/(S154C)P2–5 were further purified with ResourceQ 1 mL column (GE Healthcare) equilibrated with TEDG buffer, and eluted with a linear gradient of 0–500 mM NaCl. The fractions containing P1(Q153G)/(S154C)P2–5 dimer were collected and concentrated using Amicon Ultra-15 (Millipore). 0.2 mg of P1(Q153G)/(S154C)P2–5 was isolated. The separated unreacted P1(Q153G) and (S154C)P2–5 were reused for the next ligation reaction. In total, ~3 mg (2 mg/L culture) of the ligated product of P1(Q153G)/(S154C)P2–5 was obtained after the several repeats of ligation and purification.

For testing on-column ligation, the cell lysate of P1(Q153G) or P1(Q153) from 10 mL culture and (S154C)P2–5 from 20 mL culture were mixed together with chitin beads (450 μL of bed volume) pre-equilibrated with buffer A, and incubated at 4 °C. After washing with 4 CV of buffer A, the column was quickly soaked with 1 CV of buffer A supplemented with 1 mM EDTA and 500 mM MESNA, followed by incubation at room temperature for more than 21 h. The proteins were eluted with 4 CV of buffer A and analyzed by SDS-PAGE.

### Preparation of P12 and P3–5 domains and in vitro ligation

The plasmids encoding P12 or P3–5 domains were transformed to *E. coli* strain BL21-CodonPlus (DE3)-RP. The expression of P12 and P3–5 domains were performed as described above. The cells were harvested and re-suspended in buffer A, followed by lysis by sonication. P12(L240G) (~49 mg/L culture), P12(A241G) (~52 mg/L culture), P12(Q244G) (~57 mg/L culture), P12(G248) (~33 mg/L culture), P12(R257G) (~39 mg/L culture), (A245C)P3–5(~20 mg/L culture), and (R249C)P3–5(~10 mg/L culture) were prepared, according to the procedures for P1 and P2–5 domains. Excess molar amounts of P12(Q244G) (4 mg, a final concentration of 0.6 mM) or P12(G248) (4 mg, a final concentration of 0.6 mM) were added to (A245C)P3–5 (2 mg, a final concentration of 0.2 mM) or (R249C)P3–5 (2 mg, a final concentration of 0.2 mM), respectively. The reaction mixture was concentrated to 250 μL using centrifugal concentrators. For (A241C)P3–5, (A242C)P3–5, and (S258C)P3–5, which were cleaved during the expression, the cell lysates were directly mixed and concentrated with P12(L240G), P12(A241G), and P12(R257G), respectively. The reaction mixtures in buffer B were incubated at room temperature with gentle shaking for more than 12 h. About 1 mg of the ligated product dimers were isolated from the reaction mixture, following the same purification procedure for P1(Q153G)/(S154C)P2–5.

### Construction of plasmids for the PTS approach

The genes of *E. coli* CheA P1 (1–139) or P12 (1–238) were amplified from genomic DNA of *E. coli* strain BL21 by PCR with oligonucleotides HK845: 5′-AAC ATA TGA GCA TGG ATA TAA GCG ATT TT and HK847: 5′-AAG GAT CCG GTC ACT GCG GAT GGC GTT TC for P1, and HK845 and HK864: 5′-TAG GAT CCA AGC ACT GGT GGG GTG G for P12, respectively, and ligated into the vector pMMRSF17 (addgene ID: 20178) (Muona et al. 2008) after the digestion with *Nde*I and *Bam*HI, which resulted in pYMRSF01 and pYMRSF03, respectively. The genes of *E. coli* CheA P2–5 (147–654) or P3–5 (246–654) were amplified from genomic DNA of *E. coli* strain BL21 with oligonucleotides HK893: 5′-TTA GGT ACC AGT GAA CCG CAA GAT GAG and HK846: 5′-GTC AAG CTT AGG CGG CGG TGT TCG CCA TAC G for P2–5, and HK865: 5′-AAG GTA CCC CAA CCG GCC GCG TGG AG and HK846 for P3–5, respectively. The PCR products were ligated into pMMBAD16 (addgene ID: 20176) (Muona et al. 2008) after the digestion with *Kpn*I and *Hind*III, resulting in pYMBAD31 and pYMBAD05, respectively. For the expression of P12^(0)^ and ^(3)^P3–5 fusion proteins, the mutations were introduced at the *Bam*HI site of pYMRSF03 and the *Kpn*I site of pYMBAD05 using QuikChange^®^ protocol with oligonucleotides HK873: 5′-GCC ATC CGC AGT GAC CCG ATT ATG TTT AAG CTA TGA AAC and HK874: 5′-GTT TCA TAG CTT AAA CAT AAT CGG GTC ACT GCG GAT GGC for P12^(0)^ and HK891: 5′-CTT CTA ATT GTT TCA ATC AAG CCC CAA CCG GCC and HK892: 5′-GGC CGG TTG GGG CTT GAT TGA AAC AAT TAG AAG for ^(3)^P3–5.

The genes encoding Int_C_-P2–5 from pYMBAD31 and Int_C_-P3–5 from pYMBAD05 were ligated into the vector pHYRSF1-02 (addgene ID: 24765) (Muona et al. 2008) after the digestion with *Nde*I and *Hin*dIII, resulting in pYMRSF39 and pYMRSF38, respectively.

### In vivo PTS ligation of P1/P2–5, P12/P3–5, and mutants

*E. coli* ER2566 cells were transformed with the two plasmids encoding N-terminal fragment (H_6_-Smt3-P1-Int_N_ or H_6_-Smt3-P12-Int_N_) and C-terminal fragment (Int_C_-P2–5 or Int_C_-P3–5) and grown at 37 °C in 5 mL LB media supplemented with 100 μg/mL ampicillin and 25 μg/mL kanamycin. The first induction was induced by the addition of either IPTG to a final concentration of 0.5 mM or l-arabinose to a final concentration of 0.2% when the cell density reached an OD_600_ of 0.4−0.8. When the OD_600_ reached 1.0−1.4 after 30 min of the first induction, the second inducer was added to the cell culture at a final concentration of 0.2% l-arabinose or 0.5 mM IPTG. For the expression of either N- or C-terminal fragment, only the first induction was performed. The cells were induced at 37 °C for total 4−5 h after the first induction. The cells were pelleted by centrifugation and re-suspended with 1× SDS electrophoresis sample buffer (62.5 mM Tris (pH 6.8), 2% SDS, 10% glycerol, 0.001% bromophenol blue, and 1% 2-mercaptoethanol), and subjected to 14% SDS-PAGE analysis.

### In vivo PTS segmental isotopic labeling of [^2^H,^15^N]P12/[^1^H,^14^N]P3–5

*E. coli* ER2566 cells bearing the two plasmids pYMRSF03 and pYMBAD05 were grown at 37 °C in 1 L ^2^H_2_O M9 minimal media containing ^15^NH_4_Cl (1 g/L) and [^2^H_7_]-glucose (2 g/L) as nitrogen and carbon sources, supplemented with 100 μg/mL ampicillin, 25 μg/mL kanamycin, and Celtone-DN powder (Spectra Gases Inc., 1 g/L). H_6_-Smt3-P12-Int_N_ was first induced by the addition of IPTG to a final concentration of 0.5 mM for 13 h when the cell density reached an OD_600_ of 0.8–0.9. Subsequently, the cells were spun down for 10 min at 900×*g* and gently washed with 200 mL pre-warmed LB media containing 0.1% l-arabinose and 100 μg/mL ampicillin. After the washing step followed by centrifugation for 10 min at 900×*g* at 25 °C, the cells were re-suspended into 1,150 mL LB media containing 0.1% l-arabinose and 100 μg/mL ampicillin. Expression of Int_C_-P3–5 was induced for another 6 h at 37 °C. The cells were harvested by centrifugation and frozen at −80 °C for further purification. The purification and removal of H_6_-Smt3 were performed as described previously (Muona et al. 2008; Busche et al. 2009). The ligated product dimers were further purified by SEC and anion-exchange chromatography in the same way as in vitro EPL. 20 mg/L culture of P12/P3–5 was obtained after the final purification step.

### In vitro PTS ligation of P1/P2–5 and P12/P3–5, and segmental isotopic labeling of [^2^H,^15^N]P1/[^1^H,^14^N]P2–5

*E. coli* ER2566 cells were transformed with either plasmid of pYMRSF01 (H_6_-Smt3-P1-Int_N_), pYMRSF39 (H_6_-Int_C_-P2–5), pYMRSF03 (H_6_-Smt3-P12-Int_N_) or pYMRSF38 (H_6_-Int_C_-P3–5) and grown to mid-log phase in LB or ^2^H_2_O M9 minimal media containing ^15^NH_4_Cl (1 g/L) and [^2^H_7_]-glucose (2 g/L) as nitrogen and carbon sources and Celtone-DN powder (1 g/L). The protein expression was induced by the addition of IPTG to a final concentration of 0.5 mM. After the induction at 25 °C for 6 h, the cells were harvested and re-suspended in buffer C (20 mM sodium phosphate (pH 8.0), 300 mM NaCl). The cells were lysed by ultrasonication. The supernatant after centrifugation was subjected to purification by IMAC with HIS-Select^®^ nickel affinity gel (Sigma). After the elution with buffer C containing 100 mM imidazole, the elution fractions were dialyzed against buffer D (20 mM Tris–HCl (pH 7.5), 500 mM NaCl) at 4 °C. H_6_-Smt3-P1-Int_N_ (45 mg, a final concentration of 110 μM) or H_6_-Smt3-P12-Int_N_ (42 mg, a final concentration of 110 μM) was added to H_6_-Int_C_-P2–5 (56 mg, a final concentration of 100 μM) or H_6_-Int_C_-P3–5 (34 mg, a final concentration of 100 μM) in total volume of 10 and 7 mL, respectively. Tris(2-carboxyethyl)phosphine (TCEP) was added to a final concentration of 0.5 mM to maintain them under reducing condition. The reaction mixture was incubated at room temperature with gentle shaking for more than 12 h. The purification, removal of H_6_-Smt3, and the isolation of the ligated product dimers were performed following the same purification procedure for in vivo PTS.

### Phos-tag SDS-PAGE analyses of phosphotransfer from P1/P2–5 or P12/P3–5 to CheY

CheY, CheW, wild-type CheA, and membrane fraction containing Tar were prepared as described previously (Li and Hazelbauer 2011, and cited therein). In the preparation of membrane fraction containing Tar, the *E. coli* strain RP3098 was utilized as the host strain. Twenty picomole of the ligated CheA(P1/P2–5 or P12/P3–5) or wild-type CheA, CheW, and membrane fraction containing Tar were dissolved in 8 μL of buffer E (50 mM Tris (pH 9.0), 5 mM MgCl_2_, 50 mM KCl, and 0.5 mM DTT). The reaction mixtures were incubated at room temperature for >3 h, and 1 μL of 200 μM CheY was added to the reaction mixture. In the negative control experiment, l-aspartic acid was also added to a final concentration of 0.5 mM, which binds to Tar and inhibits the phosphorylation of CheY. The reaction mixtures were further incubated for 10 min, and the autophosphorylation was initiated by the addition of 1 μL of 30 mM ATP. After 10 s incubation, the reaction was terminated by the addition of an equivalent volume of 2×SDS electrophoresis sample buffer (125 mM Tris (pH 6.8), 4% SDS, 20% glycerol, and 0.001% bromophenol blue). For the preparation of phosphorylated CheY, 20 μM (final concentration) of CheY was dissolved in the buffer E with 10 mM acetyl phosphate (Sigma). The reaction mixtures were incubated at room temperature for 7 min, followed by addition of an equivalent volume of the 2×SDS electrophoresis sample buffer to stop the reaction. These samples were subjected to 12% Zn^2+^-, or 18% Mn^2+^-phos-tag SDS-PAGE analysis with 50 μM Phos-tag (Kinoshita et al. 2011).

## Results

**Fig. 2:**
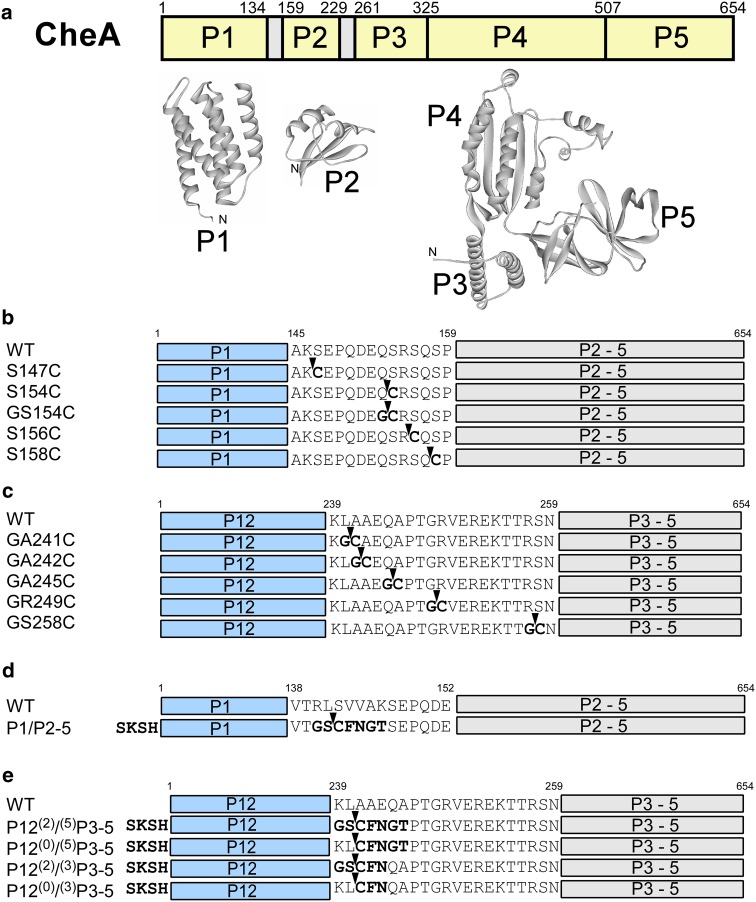
**a** Domain organization of *E. coli* CheA. The crystal structures of P1 (PDB ID: 1I5N), P2 (1FWP) and P3–5 (1B3Q) are shown in *ribbon drawing*. **b** The linker sequences between P1 and P2–5 used in EPL approach. **c** The linker sequences between P12 and P3–5 used in EPL approach. **d** The linker sequences between P1 and P2–5 used in PTS approach. **e** The linker sequences between P12 and P3–5 used in PTS approach. The wild-type linker sequences of *E. coli* CheA are shown at the top of each panel. *Arrows* indicate the ligation points. The mutations introduced for the ligation are in *bold*

*E. coli* CheA is a receptor-associated dimeric histidine kinase and one of the key components in chemotaxis signalling by autophosphorylating itself in response to chemical ligand binding to the receptors, such as Tar, and transferring the phosphoryl group to the cytoplasmic protein CheY (Baker et al. 2006; Hazelbauer et al. 2008). The structure of CheA is composed of five globular domains (P1–5) (Fig. 2a), and the dimer interface is in P3 domain. The crystal structures of P1, P2, and P3–5 domains have been determined individually, indicating that P1, P2, and P3–5 are structurally independent entities (Mourey et al. 2001; McEvoy et al. 1996; Bilwes et al. 1999). Currently, there is no structural information on the full-length protein containing all the five domains, making CheA an ideal system for NMR investigation with segmental isotopic labeling. It is also unknown how these domains are interacting with other components in chemotaxis signalling complex despite the available crystal structures (Park et al. 2006; Bell et al. 2010). Segmental isotopic labeling of one of the domains in the full-length CheA will not only facilitate NMR assignment of the full-length CheA but also open an avenue to analyse transient domain–domain interactions within CheA by transferred cross-saturation (TCS) experiments (Shimada et al. 2009). As a first step to understand the structure function relationship of CheA, we aim at establishing the labeling of either P1 or P12 domains in the full-length CheA by EPL or PTS for further investigation of the structural arrangements between each domain of CheA. The preparations by both EPL and PTS are also expected to identify the better strategy for preparing segmental labeled samples of CheA routinely.

## EPL

### Preparation of precursor proteins for chemical ligation

The first step of segmental isotopic labeling with EPL approach is to prepare two reactive fragments for in vitro chemical ligation. The N-terminal fragment requires a thioester modification at the C-terminus. The other C-terminal fragment requires an N-terminal cysteine. Although proteolytic approach is available for creating an N-terminal cysteine using Factor Xa, thorombin and others (Liu et al. 2009), we decided to use pTWIN system, which is commercially available and has been successfully used for segmental isotopic labeling (Vitali et al. 2006; Skrisovska and Allain 2008; Zhao et al. 2008). Both N- and C- precursor fragments can be conveniently produced by pTWIN vectors from the IMPACT™ kit developed by New England Biolabs (Evans et al. 1999). pTWIN vectors are attractive because it does not require any proteolytic steps to create N-terminal cysteine but also it could be directly used for “on-column” ligation (Vitali et al. 2006; Skrisovska and Allain 2008; Zhao et al. 2008; Chen and Wang 2011) (Fig. 7).

We selected four sites between the linker connecting P1 and P2 domains as the ligation sites (Fig. 2b). To minimize the structural changes due to the introduced cysteine residue required for EPL, CheA was divided into two fragments at the front of serine residue (S147, S154, S156, and S158) to replace them by cysteine. The four fragments containing residues 1–146 (P1(K146)), 1–153 (P1(Q153)), 1–155 (P1(R155)), and 1–157 (P1(Q157)) of CheA were cloned into pTWIN1 vector at the front of *Mxe* GyrA intein using the restriction sites of *Nde*I and *Sap*I sites.

Due to the mutation introduced in *Mxe* GyrA intein, the incubation of P1-intein fusion protein with a thiol agent such as MESNA releases P1 domain with α-thioester modification. However, significant amount (>50%) of P1-intein fusion protein was already cleaved off in vivo at the front of intein (N-cleavage) at 25–37 °C, releasing unmodified P1 domain (Fig. 4a; Table 1). P1(Q153) fusion construct was only the exception (Table 1). This N-cleavage considerably reduced yields of the thiol modified P1 domain, which was problematic for the next step. Lowering the expression temperature to 18 °C could suppress the undesired N-cleavage in vivo (down to about 10%) but also reduced the expression level of CheA domain-intein fusion significantly (data not shown). The other parts of P2–5, i.e. (S147C)P2–5, (S154C)P2–5, (S156C)P2–5, and (S158C)P2–5, were also cloned into pTWIN1 vector between *Sap*I and *Bam*HI sites after *Ssp* DnaB intein. Similar to the N-terminal fragments, in vivo cleavage after the intein (C-cleavage) was also observed for most of the constructs (Table 1). As the fused chitin-binding domain (CBD) was used for the purification tag, the C-cleavage lowered the recovery of the C-terminal fragments. This result indicated that pTWIN system was not the best choice for our system. The preparation of the C-terminal fragment might be possible to be improved further by using different vectors such as pTYB. Nevertheless, we obtained sufficient amounts for the second step of in vitro ligation for Q153/S154C constructs (40 mg/L culture for the N-terminal fragment and 20 mg/L culture for the C-terminal fragment) (Fig. 2b).

**Table 1:** N- and C-cleavages with pTWIN system

Split sites	N-cleavage (%) P1	C-cleavage (%) P2–5
K146/S147C	~50	~100
Q153/S154C	~10	~50
R155/S156C	~50	~90
Q157/S158C	~40	~0

### In vitro chemical ligation of P1/P2*–*5

The purified P1(Q153) with C-terminal thioester modification (10 mg in a final concentration of 0.8 mM) and (S154C)P2–5 with an N-terminal cysteine (4 mg in a final concentration of 0.1 mM) were reacted in vitro in the presence of 200 mM MESNA at pH 7.4 for 2–5 days. Even though the ligation product of P1/P2–5 was observed, the ligation efficiency was disappointingly low (<10%) (Fig. 4c, lanes 1–4). Furthermore, dimer formation between the ligated CheA and the unreacted P2−5 further reduced the final yield of the P1/P2–5 dimer during the purification (less than 0.1 mg/L). In the following context, we refer to the ratio of the ligated P1/P2–5 to the substrate P2–5 monomers as “ligation efficiency”, and the amount of the purified dimer product (i.e., dimeric P2–5s ligated with two P1s), as “dimer yield”. The longer incubation did not improve the ligation efficiency (Fig. 4c). We also tested “on-column” ligation of the two fragments on the chitin beads (Vitali et al. 2006; Skrisovska and Allain 2008; Chen and Wang 2011). However, we could not improve the ligation efficiency significantly with “on-column” protocol, suggesting that the local reactant concentrations on the column might be still insufficient for the ligation of CheA by EPL. The importance of amino-acid types of C-terminal residue with the thioester modification for NCL has been previously reported, pointing out that smaller side-chains are preferable for improving the efficiency (Hackeng et al. 1999; Johnson and Kent 2006). Once thioester modification is introduced after the release from the intein-fusion by thiol agents, EPL is identical to NCL. To improve ligation efficiency, we introduced a mutation of Q153G at the C-terminus of P1 domain, i.e. P1(Q153G), which had negligible N-cleavage during the expression. Even though Gly was not previously recommended for *Mxe* GyrA intein, we could efficiently cleave P1 domain by MESNA in vitro (Liu et al. 2009). The chemical ligation efficiency between P1(Q153G) and (S154C)P2–5 was improved to about 40% (Fig. 4c, lane 5), which is significantly better than the ligation efficiency between P1(Q153) and (S154C)P2–5. This result confirms that the choice of the amino-acid type at the C-terminus is of critical importance for EPL approach. However, we were unable to improve the ligation efficiency further by longer incubation, changing protein concentrations, varying MESNA concentrations (20–200 mM), or additions of 4-mercaptophenylacetic acid (MPAA), TCEP, or 0.2 M guanidine hydrochloride. Only the ligation kinetics was improved in the presence of MPAA (data not shown). The unreacted P1(Q153G) was removed by size exclusion chromatography (SEC), and the unreacted (S154C)P2–5 were further removed by anion exchange chromatography. Dimer formation of the ligated product was evident from the SEC profiles with a similar elution volume as immunoglobulin G (158 kDa) (Supplemental Figs. 2a, c). CheA requires the dimer formation for the functionality (Surette et al. 1996). The isolated Q153G/S154C dimer was active in phosphorylation of CheY in the presence of receptor protein Tar, adaptor protein CheW, and ATP as phosphorylation of CheY as observed by phos-tag™ SDS-PAGE analysis (Supplemental Fig. 1a). The activity of the ligated products also supports the functional dimer formation. We could obtain about 0.2 mg of the ligated CheA of Q153G/S154C dimer from one litre culture for P1(Q153G) (~11 mg of P1(Q153G) as the starting material). The isolated unreacted P1 and P2–5 fragments from the reaction mixture still retained ligation activity. It should be noted that the heterodimer between unreacted (S154C)P2–5 and the ligated product was also present due to the P3 domain responsible for the dimerization. Although the heterodimer could be separated by the following anion exchange chromatography, heterodimer formation with unreacted precursors complicated the subsequent purification and reduced the final yield of the ligated product homodimer (The dimer yield is theoretically proportional to the square of the ligation efficiency). We had to use the excess of the labeled material of P1(Q153G) to facilitate the subsequent separation of the ligated homodimer from the heterodimer between the ligated product and the reactant. The sufficient CheA dimer for an NMR sample was attained only by repeating the ligation reaction a few times with the re-purified unreacted fragments retaining the reactivity. This is not a typical EPL procedure, but we could efficiently utilize the labelled material. [^1^H,^15^N]-TROSY spectrum of the [^2^H,^15^N]-P1/[^1^H,^14^N]-P2–5 clearly indicates the reduction and improvement of NMR signal overlaps compared with the uniformly labeled sample (Figs. 6a, b).

### Preparation of the two fragments for the ligation P12/P3–5 by EPL

Next, we tested the ligation between P12 and P3–5. We chose five split sites for the ligation (Fig. 2c). Based on the results from the P1/P2–5 ligation, the C-terminal residue was replaced by glycine residue to improve the ligation efficiency. All the fusion protein with *Mxe* GyrA intein showed only slight N-cleavage (<10%), making these constructs suitable for the purification with chitin column (data not known). However, the preparations of the C-terminal fragment with an N-terminal cysteine still suffered from C-cleavages (Table 2). P3–5 with A245C and R249C mutations had sufficiently low C-cleavages for further purification. The N-terminal CBD domain for purification was not practical due to this C-cleavage in the most of the cases, requiring some optimizations. Therefore, it might be more predictable to use proteases for introducing an N-terminal cysteine rather than pTWIN system.

**Table 2:** The yields of the C-terminal fragment (P3–5) with pTWIN system

Split sites	C-cleavage (%) P3–5	Yields (mg/L)
A241C (A)	~80	n.a.
A242C (E)	~100	n.a.
A245C (P)	~20	~60
R249C (V)	~50	~30
S258C (N)	~90	n.a.

Amino-acid types of the following residues are shown in brackets

*n.a.* not available

### In vitro chemical ligation of P12/P3*–*5

In vitro ligation of pairs of the two precursor fragments, i.e. Q244G/A245C (4/2 mg) and G248/R249C (4/2 mg), was performed in 250 μL volume of buffer B containing 200 mM MESNA at room temperature. Only two pairs (Q244G/A245C and G248/R249C) could be tested with the purified components due to the cleavages. The ligation efficiency was about 30% from the estimation of SDS-PAGE. We also tested if crude extracts could be used for the ligation of the other combinations, which is not usually done with EPL approach. As shown in Fig. 4d, the ligated products were detected in the most of the combinations. The best ligation results were for the purified Q244G/A245C and G248/R249C combinations, suggesting that crude extract cannot be efficiently used for the ligation unlike PTS approach. Both Q244G/A245C and G248/R249C were further purified by SEC and anion exchange chromatography to remove unreacted precursors. The typical yields were about 1 mg from one litre culture for P12.

## PTS

### In vivo ligation of P1/P2–5 and P12/P3–5

**Fig. 3:**
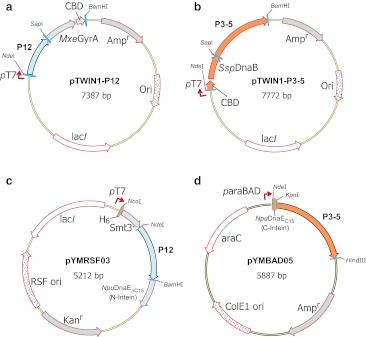
Vector maps used with EPL (**a** and **b**) and PTS approaches (**c** and **d**). **a** P12 domains were cloned at the front of *Mxe* GyrA intein fused with CBD in pTWIN1 vector. **b** P3–5 domains were cloned after *Ssp* DnaB intein fused with CBD domain in pTWIN1 vector. **c** P12 domains were cloned at the front of N-intein in pMMRSF17 as a fusion protein with yeast Smt3 protein. **d** P3–5 domains were cloned after C-intein in pMMBAD16

For segmental isotopic labeling with PTS, there are two strategies. One is to use artificially split inteins that require refolding steps in vitro such as PI-*Pfu*I intein (Yamazaki et al. 1998; Otomo et al. 1999a; Yagi et al. 2004). The other is to use naturally occurring split inteins without any refolding steps. As P1 and P2 domains can be detached from the rest of the protein and are therefore structurally independent entities, it should be feasible to perform protein ligation by PTS without any refolding processes (Mourey et al. 2001; McEvoy et al. 1996; Bilwes et al. 1999). We therefore decided to use the dual vector system exploiting naturally split DnaE intein from *Nostoc punctiforme* (*Npu*), which we have previously developed (Züger and Iwai 2005; Muona et al. 2010). In particular, we used an engineered naturally occurring split DnaE intein, which has a shorter C-terminal fragment consisting of only 15 residues and is less disturbing the solubility of fused proteins (Muona et al. 2008; Aranko et al. 2009). Unlike EPL approach, only one split site was selected for each ligation, namely, after residue L141 between P1 and P2 domains and after residue L240 between P2 and P3 domains as the ligation sites (Figs. 2d, e). These locations were chosen because of long linker between these domains. The choice of the split site can be crucial for PTS. Seven residues (GSCFNGT) were introduced between the N- and C-terminal fragments (Figs. 2d, e). The use of the cloning sites such as *Kpn*I and *Bam*HI in the vectors resulted in introducing sequences of GS and GT in the protein. In addition, we introduced the natural C-extein sequence of “CFN” to minimize the unfavourable effect from the junction sequences on the splicing efficiency but the length of connecting linker was kept as the original linker. The N-terminal precursor was cloned under an inducible T7 promoter with IPTG (Fig. 3c). In addition, we used N-terminally hexahistidine (H_6_) tagged Smt3 protein as an N-terminal fusion protein for convenient purification (Muona et al. 2008). The C-terminal precursor bearing C-intein was cloned into a compatible plasmid with inducible arabinose promoter (Fig. 3d). These two plasmids contain compatible origins, i.e. ColE1 and RSF origins, enabling the dual expression in the same cells. Each precursor protein in *E. coli* cells harbouring the two plasmids can be induced by addition of either IPTG or arabinose (Fig. 5a, lanes 1 or 2). Dual induction with both IPTG and arabinose induces protein *trans*-splicing in vivo, producing a larger protein because of the ligation between H_6_-Smt3-P1 and P2–5 (Fig. 5a, lanes 3 and 4). Similarly, the ligation of P12 and P3–5 by PTS was observed after 2 h of the dual expression (Fig. 5c, lanes 2 h). In both cases, one of the precursor proteins was nearly completely processed, indicating that the ligation was not limited by the splicing reaction but supply of the precursors. 2–20 mg of the ligated product can be purified from 1 L culture.

### Optimization of the linker sequences

In the PTS, we introduced a sequence of “GSCFNGT” instead of “KLAAEQA” in the linker between P2 and P3 domains. In contrast to EPL with which 1–2 residues were mutated, 7-residue change is relatively long and might have introduced functional and structural changes in the ligated protein as compared with the wild-type. We constructed the N-terminal precursor in which the N-terminal junction GS was mutated to KL (referred to as P12^(0)^) and the C-terminal precursor in which “GT” was mutated to “QA” (referred to as ^(3)^P3–5) to minimize the mutations in the linker region. All four combinations of the N- and C-precursors were tested (Fig. 5b). The “GSCFNGT” construct of P12^(2)^/^(5)^P3–5 appeared to show the strongest ligation band. Changing either the N-terminal 2 residues, the C-terminal 2 residues, or the both sides had little effect on the yields (3.6 mg/L for P12^(2)^/^(5)^P3–5, 3.5 mg/L for P12^(2)^/^(3)^P3–5, 2.5 mg/L for P12^(0)^/^(5)^P3–5, and 3.1 mg/L for P12^(0)^/^(3)^P3–5 after the purification). P12^(2)^/^(5)^P3–5 gave the best yield. We also confirmed the activities of all the ligated products of P12^(2)^/^(5)^P3–5, P12^(2)^/^(3)^P3–5, P12^(0)^/^(5)^P3–5, and P12^(0)^/^(3)^P3–5 (Supplemental Fig. 1b).

**Fig. 4:**
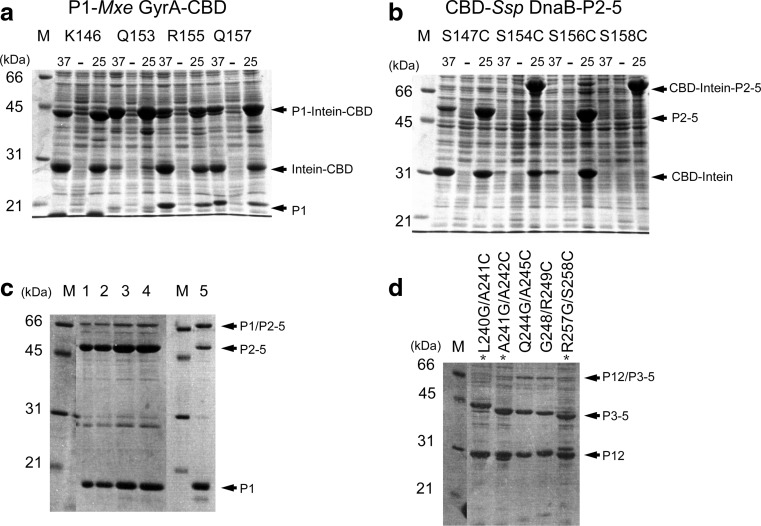
Protein ligation by EPL. **a** Expression of P1-intein fusion proteins at various split sites for the preparation of the N-terminal fragment. 37 and 25 indicate the samples expressed at 37 and 25 °C, respectively. “-“ indicates before the induction. **b** Expression of intein-P2–5 fusion proteins at various split sites for the preparation of the C-terminal fragment bearing an N-terminal cysteine. **c** *Lanes 1–4*: in vitro protein ligation of P1(Q153) and (S154C)P2–5 under several conditions. *Lane 1*: after 2 days at r.t., *lane 2*: after 5 days at r.t., *lane 3*: additional 12 h at 24 °C incubation after 2 days at r.t., *lane 4*: additional 12 h incubation at 30 °C after 2 days at r.t., *lane 5*: in vitro protein ligation of P1(Q153G) and (S154C)P2–5 after 3 days incubation at r.t. **d** In vitro protein ligation of P12 and P3–5 with various ligation sites after 15 h incubation. The ligation sites are indicated at the top of lanes. *Asterisks* indicate the reaction mixtures with crude extracts

**Fig. 5:**
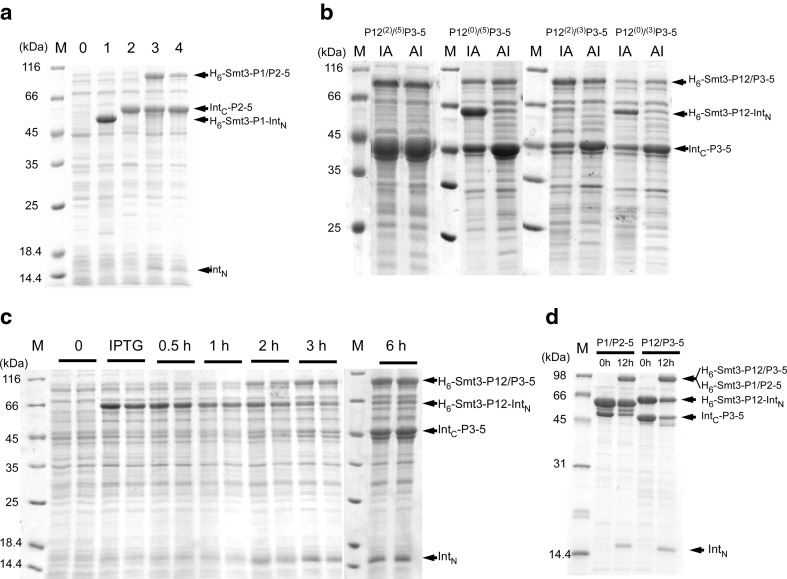
Protein ligation by PTS. **a** In vivo protein ligation of P1 and P2–5. *Lane M*: Protein marker, *lane 0*: before induction, *lane 1*: after IPTG induction, *lane 2*: After induction with arabinose, *lane 3*: Induction with IPTG followed by arabinose, *lane 4*: Induction with arabinose followed by IPTG induction. **b** The effect of the ligation junction between P12 and P3–5. *AI* indicates the samples in which the cells were induced first with arabinose for 0.5 h followed by IPTG induction for additional 3–5 h. *IA* indicates the samples in which the cells were first induced with IPTG for 0.5 h and followed by induction with arabinose for additional 3–5 h. **c** Time course of in vivo protein ligation of P12/P3–5. *Lanes 0*: before induction, *lanes IPTG*: 13 h after IPTG induction, *lanes 0.5* *h*: 0.5 h after addition of arabinose, *lanes 1* *h*: 1 h after arabinose addition, *lanes 2* *h*: 2 h after arabinose induction, *lanes 3* *h*: 3 h after arabinose induction, *lanes 6* *h*: 6 h after arabinose induction. **d** In vitro protein ligation of P1/P2–5 and P12/P3–5. *Lanes 0* *h*: before incubation, *lanes 12* *h*: after 12 h incubation at room temperature. Int_N_ and Int_C_ stand for N-intein and C-intein respectively

### Production of [^2^H,^15^N]-P12/[^1^H,^14^N]-P3*–*5 in vivo

For the preparation of segmental isotope-labeled samples of P12/P3–5, we decided to keep the seven-residue change in the linker to obtain the highest yield. We used the time-delayed dual expression approach for segmental isotopic labeling, which was previously described (Züger and Iwai 2005; Muona et al. 2010). In this in vivo protocol, one of the precursors was first expressed in the labeled medium and followed by the medium exchange and the second precursor induction. We aimed at incorporating (^2^H,^15^N) in the P12 domains and retaining the other domains unlabeled, i.e. (^1^H,^14^N).

**Fig. 6:**
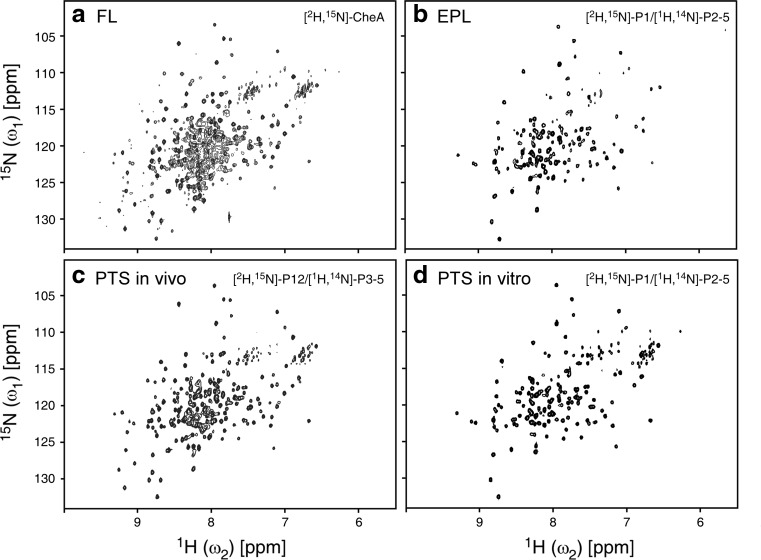
**a** ([^1^H,^15^N])-TROSY spectrum of 150 μM uniformly ([^2^H,^15^N])-labeled CheA recorded with 800 MHz spectrometer at 30 °C. **b** ([^1^H,^15^N])-TROSY spectrum of 12 μM segmentally ([^2^H,^15^N])-P1/([^1^H,^14^N])-P2–5 labeled CheA by EPL recorded with 600 MHz spectrometer at 30 °C. **c** ([^1^H,^15^N])-TROSY spectrum of 150 μM segmentally ([^2^H,^15^N])-P12/([^1^H,^14^N])-P3–5 labeled CheA by PTS in vivo recorded with 800 MHz spectrometer at 30 °C. **d** ([^1^H,^15^N])-TROSY spectrum of 100 μM segmentally ([^2^H,^15^N])-P1/([^1^H,^14^N])-P2–5 labeled CheA by PTS in vitro recorded with 800 MHz spectrometer at 30 °C

The time course of in vivo ligation for segmental isotopic labeling is shown in Fig. 5c. In the labeled perdeuterated medium containing ^15^N, the N-terminal precursor was induced for 13 h by the addition of IPTG. The induced cells were harvested with brief centrifugation and re-suspended with unlabeled LB medium containing arabinose for the induction of the C-terminal precursor bearing P3–5 domains for another 6 h induction. The dual expression took total 19 h. The ligated product was further purified by immobilized-metal ion affinity chromatography (IMAC) via the N-terminal H_6_-tag, and the unreacted P12^(2)^ and ^(5)^P3–5 were removed by SEC and anion exchange chromatography. We also confirmed the dimer formation of P12^(2)^/^(5)^P3–5 by SEC (Supplemental Figs. 2b and 2c). The fused H_6_-Smt3 was removed by digesting with Ubiquitin-like-specific protease (Ulp1) and IMAC. We could obtain 20 mg/L culture of ligated CheA dimer, quantified from the optical density at 280 nm. It is noteworthy that the high ligation efficiency in vivo diminished unfavourable heterodimer formation between unreacted P3–5 and the ligated product. ([^1^H,^15^N])-TROSY spectrum is in agreement with ([^2^H,^15^N])-P1/[^1^H,^14^N]-P2–5 prepared by EPL with additional signals that are presumably derived from P2 domain (Figs. 6a–c). The ligation and labeling was also confirmed by MALDI-TOF mass spectrometry (expected 73,565 dalton, measured 73,219 dalton). However, we also detected fully unlabeled form in the purified fraction judging from the mass spectrum and 1D NMR spectrum, which was invisible in the TROSY spectrum and estimated to be about 50% (Supplemental Fig. 3). This is due to the fact that we used the T7 promoter for the first induction, which was not shut down by changing the medium even with an additional washing step (Züger and Iwai 2005; Muona et al. 2010).

### Production of ([^2^H,^15^N])-P1/[^1^H,^14^N]-P2–5 in vitro

The low labelling selectivity observed with the in vivo prepared sample prompted us to test in vitro PTS approach. The two precursor fragments were expressed and purified separately for *trans*-splicing by mixing them in vitro. Although both N- and C-terminal fragments must be soluble for the purification, there is no isotope-scrambling between them in in vitro PTS (Muona et al. 2010). We prepared additional H_6_-Int_C_-P2–5 and H_6_-Int_C_-P3–5 constructs, where an additional H_6_-tag was incorporated at the front of C-intein for IMAC purification. Purified H_6_-Smt3-P1-Int_N_ (70 mg/L culture), H_6_-Smt3-P12-Int_N_ (100 mg/L culture), H_6_-Int_C_-P2–5 (150 mg/L culture), and H_6_-Int_C_-P3–5 (200 mg/L culture) were prepared separately. P1 (45 mg, 110 μM) and P2–5 (56 mg, 100 μM) or P12 (42 mg, 110 μM) and P3–5 (34 mg, 100 μM) were mixed in the presence of 0.5 mM TCEP at room temperature in total volume of 10 and 7 mL, respectively. SDS-PAGE analyses confirmed that both P1/P2–5 and P12/P3–5 ligated products were formed after 12 h with over 70% ligation efficiency (Fig. 5d). H_6_-Smt3 tag of the ligated product was digested and the unreacted fragments were removed by the same purification procedure as in vivo PTS. The yields were 3.6 mg/L for P1/P2–5 and 7.5 mg/L for P12/P3–5.

Relatively higher yield of CheA enabled us to yield sufficient amount of the dimer for NMR samples without repeating the reaction and purification. In the [^1^H,^15^N]-TROSY spectrum of ([^2^H,^15^N])-P1/[^1^H,^14^N]-P2–5 prepared by in vitro PTS (Fig. 6d), the signals were in good agreement with those from EPL (Fig. 6b), except for several signals, which are presumably from P1 to P2 linker region.

## Discussion

We successfully used both EPL and PTS as protein ligation methods to produce a full-length active dimeric CheA to prepare segmentally isotope-labeled CheA. The biggest challenge in producing segmentally isotope-labeled samples for both approaches is to obtain sufficient amounts of ligated products for NMR studies. This is particularly important for larger proteins because ^2^H labeling is essential for large proteins, therefore requiring high yields from smaller cultures. Optimizations at each step in EPL approach were required for our preparation of NMR samples of CheA. Both (1) good production protocols for N- and C-terminal precursors and (2) high ligation efficiency between them are essential for obtaining sufficient amount for NMR. The latter is especially critical especially for multimeric proteins like CheA, because yields of multimers are proportional to the powers of the ligation efficiency. We could achieve only 10–40% ligation efficiency for CheA with EPL, reducing the yields of the ligated homodimer significantly. We could not improve the final yield further by optimizing the reactant concentrations (0.1–1.5 mM) or adding other known reducing agents that could improve the ligation such as TCEP and MPAA (Johnson and Kent 2006). It is also noteworthy that reactant concentrations typically used for successful ligation using NCL (1–5 mM), which is the ligation mechanism of EPL, is difficult to achieve under non-denaturing conditions particularly for larger proteins like CheA (This concentration corresponds to 70–350 mg/mL for CheA)(Hackeng et al. 1999).

**Fig. 7:**
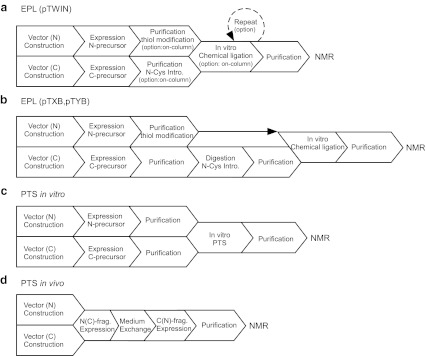
Flow charts of the procedures for preparing segmental isotopic labeled samples. **a** EPL with pTWIN system, **b** EPL with pTXB or pTYB vectors, **c** PTS in vitro, and **d** PTS in vivo

pTWIN system can introduce an N-terminal cysteine residue without any proteolytic processing and allows us to perform on-column ligation (Vitali et al. 2006; Skrisovska and Allain 2008; Zhao et al. 2008; Chen and Wang 2011). However, the use of pTWIN system often resulted in undesired N- and C-cleavages in vivo. In this case, purification via CBD fused with the intein was not ideal like our case of CheA. An additional purification tag or proteolytic approach might be more useful and reliable although it introduces additional steps prior to the ligation reaction (Fig. 7b). For the preparation of the N-terminal precursor, in vivo N-cleavage was more problematic because the intein-mediated thioester modification is no longer possible when the precursor protein is cleaved off in vivo. N-cleavage is also dependent on the preceding residue of *Mxe* GyrA intein, restricting the split site for ligation (Southworth et al. 1999). Although the previous report suggested that Lys, Arg and Gln at the C-terminus did not induce significant N-cleavages at 37 °C, considerable cleavages were observed with these residues between P1s and *Mxe* GyrA intein. Therefore, it was essential to optimize the split sites or conditions with which in vivo N-cleavage could be sufficiently suppressed for CheA constructs. Moreover, the C-terminal residue with the thioester modification plays an important role in achieving high ligation efficiency as reported previously (Hackeng et al. 1999; Johnson and Kent 2006). We observed the improvement from 10% to about 40% by replacing the C-terminal residue of P1 from glutamine to glycine. In theory, EPL requires only one residue of cysteine in the sequence at the ligation junction. In the practical case of CheA, the choice of the ligation site might define the yield by EPL although the EPL conditions used in this study can still be improved further to increase the yields with more extensive optimizations at each step. We speculate that lower ligation efficiency could be due to some structural features of the CheA dimer that interferes with the ligation reaction and/or insufficient reactant concentrations. Thus, not all split sites in CheA can be ligated with high efficiency, constraining the split sites. EPL approach might be more powerful when the protein needs to be refolded, because NCL can be performed under denaturing conditions unlike PTS, which requires a proper folding of the intein. EPL approach with CheA had a large array of parameters at each step to optimize such as temperature, incubation time, split sites, protein concentrations, and so on. These parameters seem to be protein-specific and requiring time-consuming optimizations for individual cases (Zhao et al. 2008; Clerico et al. 2010; Karagöz et al. 2011).

PTS approach with CheA required much less optimization because PTS with the initially tested split site for each linker worked well. Because of the high ligation efficiency with both in vivo and in vitro ligation of CheA by PTS, both P1/P2–5 and P12/P3–5 could be ligated with reasonable yields under a standard condition without any further optimization (2–20 mg/L). This is presumably because PTS works under similar conditions to the physiological condition and the affinity between split intein fragments is very high (K_D_ = 3 nM), thereby less depending on the reactant concentrations (Shah et al. 2011). The ligated product of CheA could be produced in vivo within one day in *E. coli* by PTS, requiring much shorter preparation time than EPL approach (total >7 days even after the optimization). The ligated product was further purified with the incorporated removable tag. The in vivo PTS approach was simpler with CheA because it was not necessary to purify the two individual precursors separately (Fig. 7d). One of the disadvantages of in vivo protocol is that stable isotopes can be carried over to the second expression after the medium exchange, reducing the selectivity of the labeling. This isotopic scrambling can be reduced by optimizing the order of the expression and extra washing steps (Muona et al. 2010). Depending on the applications of segmentally isotope-labeled samples, one must pay attentions to the selectivity of isotopic labeling while establishing an in vivo protocol. When the low labeling selectivity of the in vivo protocol is problematic, in vitro PTS approach might be a more promising alternative as there is no isotope scrambling (Fig. 1b) (Muona et al. 2010). While the labeling selectivity of in vitro PTS method is as high as that of EPL, ligation efficiency was higher without any optimization, which enabled us to prepare NMR samples of CheA with high labeling selectivity in shorter preparation time (Table 3 and Fig. 7). Soluble precursor fragments with efficient splicing junction sequences, in which split intein fragments are fused, are prerequisite for the PTS approach, particularly for in vitro PTS (Muona et al. 2010). In the case of CheA, such fragments were obtained without extensive optimization presumably because CheA is composed of five self-contained domains and long linkers, facilitating the introduced non-native junction sequence without loss of the function. In the case of multi-domain proteins with short linkers, optimization of the split site could be necessary for accommodating the introduced sequence as “scar” at the ligation site. On the other hand, EPL approach requires fewer mutations at the ligation site. It is also applicable for insoluble precursor fragments as the ligation can be performed under denaturing conditions even though the protein must be refolded after the ligation. In both EPL and PTS approaches, it is likely to introduce some changes in the protein sequence around the ligation site unless the target protein contains an ideal sequence at the targeted site for ligation. Therefore, functional assay is probably essential to verify the functionality of the ligated protein with the introduced mutations. In the future, it might be possible to use different robust and promiscuous split inteins with different splicing junction sequences that could reduce changes around ligation sites (Ellilä et al. 2011).

**Table 3:** Summary of EPL and PTS results with CheA

	EPL	PTS in vivo	PTS in vitro
Sample preparation time	>7 days	2–3 days	4–5 days
Ligation efficiency	<40%	~100%^a^	~70–80%
Number of split site mutants	5	1	1
Labeling selectivity	High	Low^b^	High
Reactant concentrations (Recommended conc.)	0.2–1.5 mM (1–5 mM)	Unknown –	~0.1 mM (>10 nM or less)^c^
Number of mutated residues	1–2	7^d^	7^d^

^a^Only the ligated product was purified from the bacterial cells

^b^Due to the induction order of IPTG followed by arabinose

^c^The minimally required concentration for PTS estimated from the affinity between N- and C-intein of *Npu* DnaE intein

^d^Three-residue mutation may be applicable with slightly reduced yields

In summary, functional full-length CheA suitable for NMR studies could be produced by both EPL and PTS approaches. This study highlights that the current problem in segmental isotopic labeling is to achieve high ligation efficiency at desired locations and that the choice of the ligation site is crucial for both methods. EPL approach was more difficult to achieve high ligation efficiency for CheA mainly because of typically high reactant concentrations required for NCL and the dimeric nature of CheA (Table 3). PTS approach currently may require a longer sequence alternation to retain high ligation efficiency. It is also necessary to verify biological functions of ligated products prepared by EPL or PTS to ensure that changes in the ligation region do not alter their functionalities. In the case of 140 kDa dimeric CheA, PTS approach was much less time-consuming than EPL for the preparation because of a smaller number of parameters to be optimized as the sequence change introduced around the ligation site was acceptable for the function (Table 3 and Fig. 7). This could be simply due to the fortunate design of the initial constructs and the long linkers in CheA. Unfortunately, there is presently no known efficient intein that can perform PTS at any junction sequences with any proteins. Thus, PTS currently requires at least a few or more mutations near the ligation site, limiting the applicability of PTS. Such promiscuous and robust split inteins, if any, could be used for traceless ligation at various ligation sites and could make PTS approach more general. For EPL, a simple and reliable way to produce a thioester-modified precursor fragment is desirable to reduce the optimization steps without testing various split sites. A general, simple, and robust approach for segmental isotopic labeling remains to be further developed to facilitate routine NMR studies of large proteins.

## Electronic supplementary material

Below is the link to the electronic supplementary material.

Figure S1: Activity tests of the ligated CheA by phosphate-affinity SDS-PAGE. Phosphorylation assays using the ligated CheA produced by EPL (a) and by PTS* in vivo* and* in vitro* (b). Results of the four variants of P12/P3−5 produced by PTS* in vivo* and P1/P2−5 produced by PTS* in vitro* with 7-residues mutation are shown. Sequences of the ligation sites are indicated at the top of the lanes. pCheY is phosphorylated CheY produced by the ternary complex of CheW, CheA, and Tar, confirming the activity of CheA. Asp (aspartic acid) is the ligand of the receptor Tar, which inhibits phosphorylation of CheY. AcPh (acetyl phosphate) is a small molecule that phosphorylates CheY* in vitro*. (TIFF 316 kb)

Figure S2: SEC profiles of the ligation reaction mixtures of P1/P2–5 produced by (a) EPL or (b) PTS in vivo, and (c) molecular weight markers (immunoglobulin G (IgG) and bovine serum albumin (BSA)). Arrows indicate peaks containing the ligated P1/P2–5, IgG, and BSA. (TIFF 316 kb)

Figure S3: MALDI-TOF mass spectrometry analyses of (a) P12/P3–5 prepared by PTS in vivo and (b) P1/P2–5 prepared by PTS in vitro*.* (TIFF 180 kb)
