# Supplementary figures and images for: Segmental isotopic labeling of a 140 kDa dimeric multi-domain protein CheA from Escherichia coli by expressed protein ligation and protein trans-splicing
Source: J Biomol NMR. 2012 Jun 28;53(3):191–207. doi: 10.1007/s10858-012-9628-3 (PMC3405243; doi:10.1007/s10858-012-9628-3)

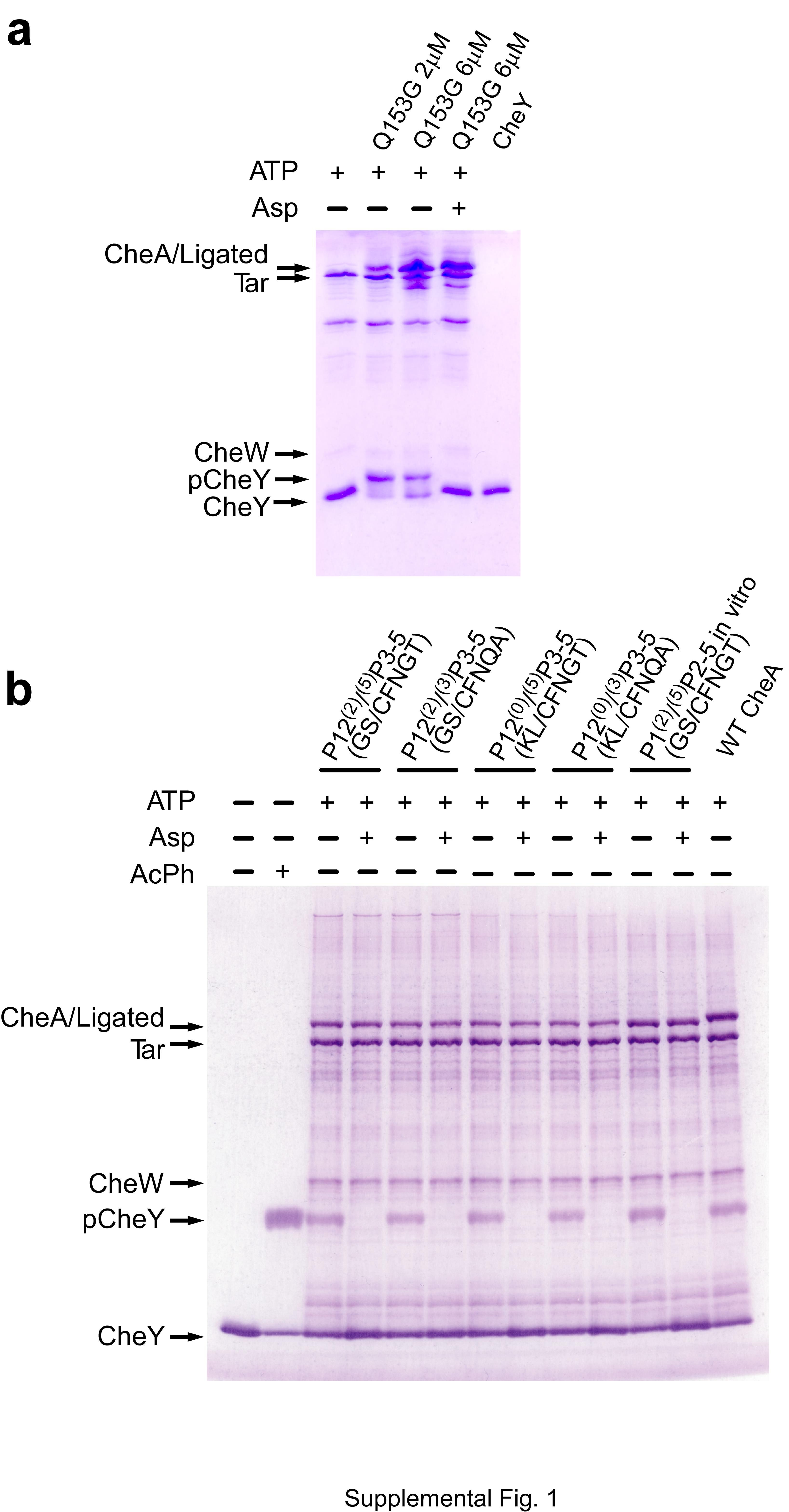

Supplement: Supplementary file 1 — Figure S1: Activity tests of the ligated CheA by phosphate-affinity SDS-PAGE. Phosphorylation assays using the ligated CheA produced by EPL (a) and by PTS in vivo and in vitro (b). Results of the four variants of P12/P3−5 produced by PTS in vivo and P1/P2−5 produced by PTS in vitro with 7-residues mutation are shown. Sequences of the ligation sites are indicated at the top of the lanes. pCheY is phosphorylated CheY produced by the ternary complex of CheW, CheA, and Tar, confirming the activity of CheA. Asp (aspartic acid) is the ligand of the receptor Tar, which inhibits phosphorylation of CheY. AcPh (acetyl phosphate) is a small molecule that phosphorylates CheY in vitro. (TIFF 316 kb) [file 10858_2012_9628_MOESM1_ESM.jpg]

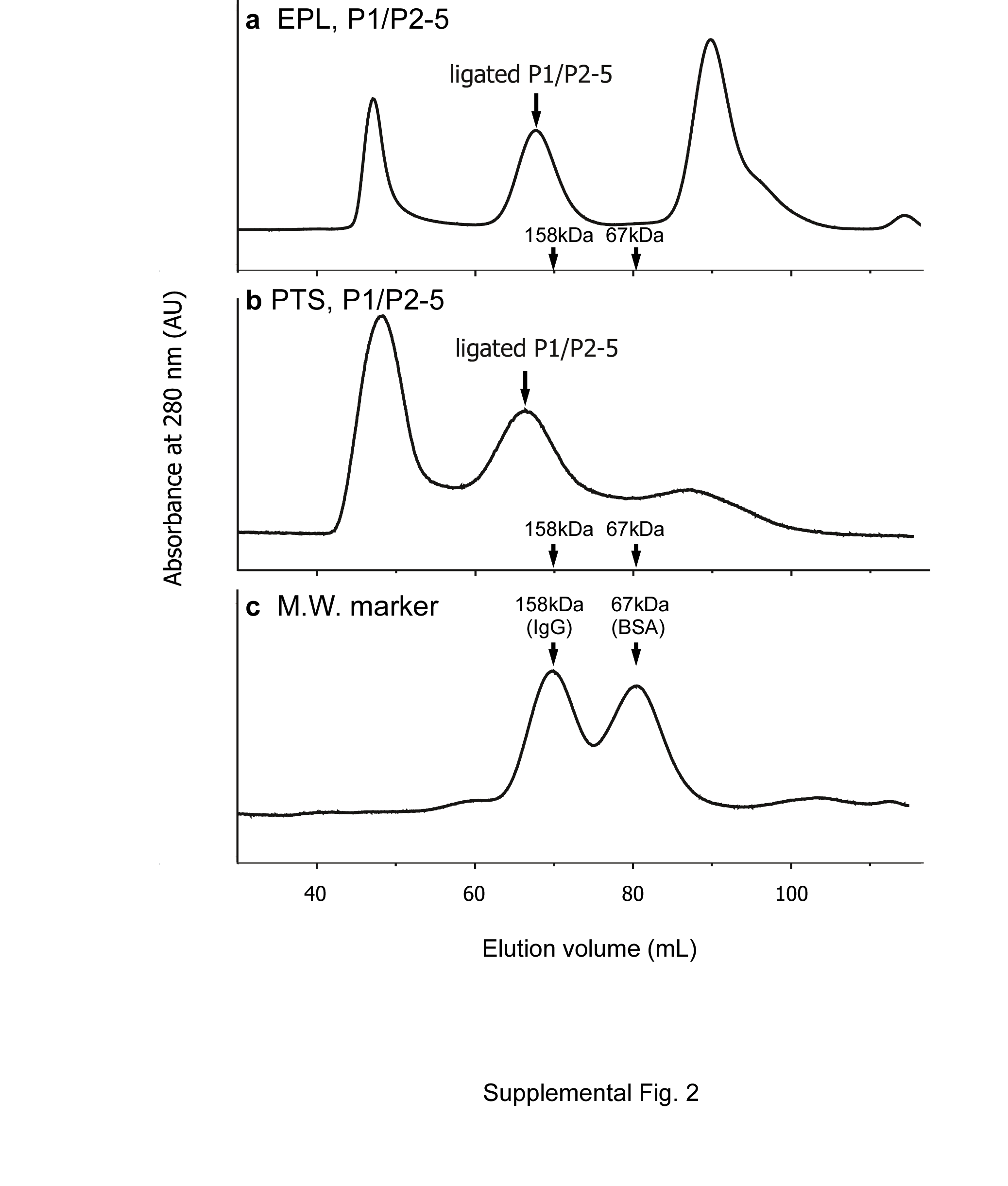

Supplement: Supplementary file 2 — Figure S2: SEC profiles of the ligation reaction mixtures of P1/P2–5 produced by (a) EPL or (b) PTS in vivo, and (c) molecular weight markers (immunoglobulin G (IgG) and bovine serum albumin (BSA)). Arrows indicate peaks containing the ligated P1/P2–5, IgG, and BSA. (TIFF 316 kb) [file 10858_2012_9628_MOESM2_ESM.tif]

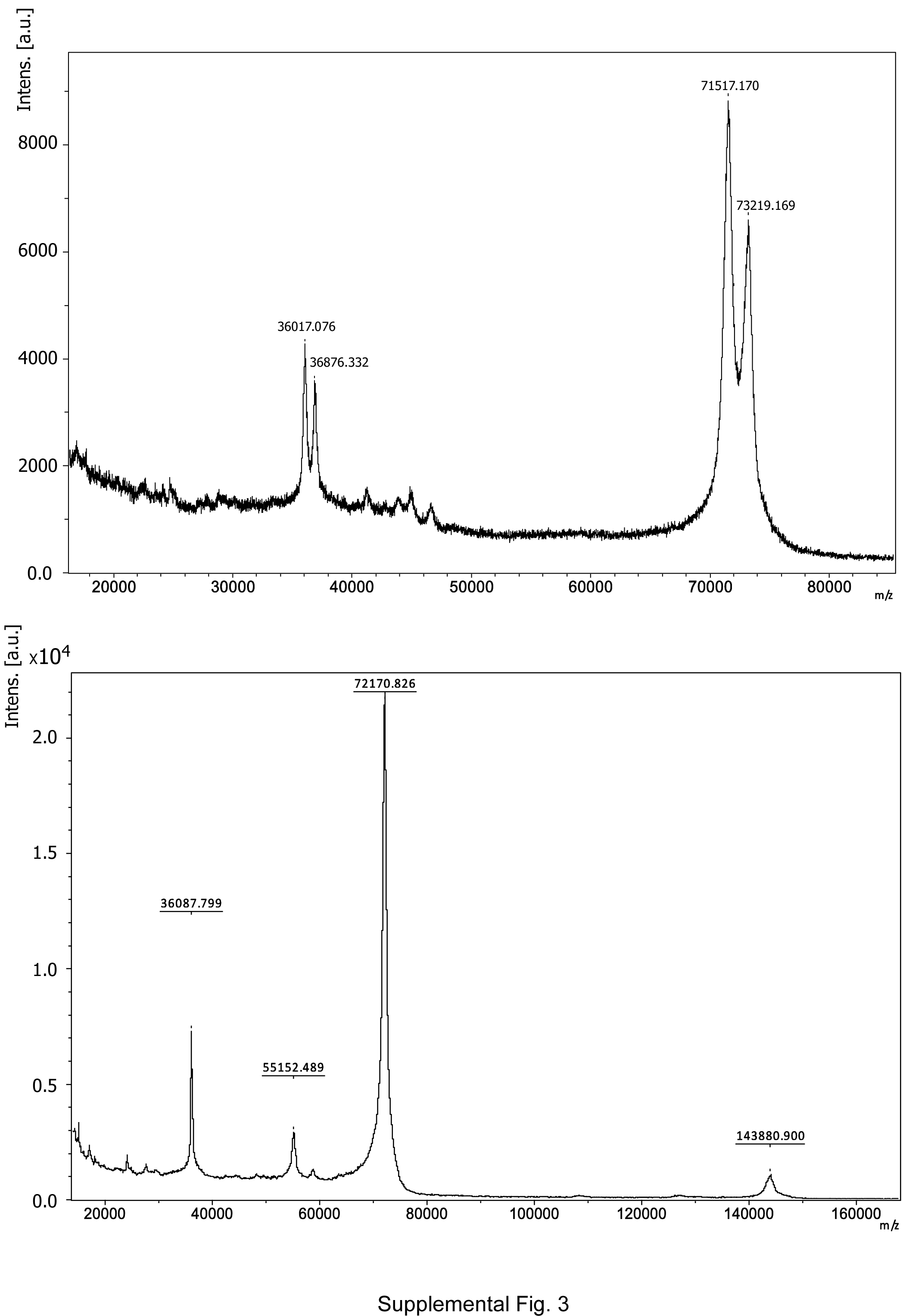

Supplement: Supplementary file 3 — Figure S3: MALDI-TOF mass spectrometry analyses of (a) P12/P3–5 prepared by PTS in vivo and (b) P1/P2–5 prepared by PTS in vitro. (TIFF 180 kb) [file 10858_2012_9628_MOESM3_ESM.tif]
